# Supplementary figures and images for: Predictors of mental health during the Covid-19 pandemic in the US: Role of economic concerns, health worries and social distancing
Source: PLoS One. 2020 Nov 11;15(11):e0241895. doi: 10.1371/journal.pone.0241895 (PMC7657497; doi:10.1371/journal.pone.0241895)

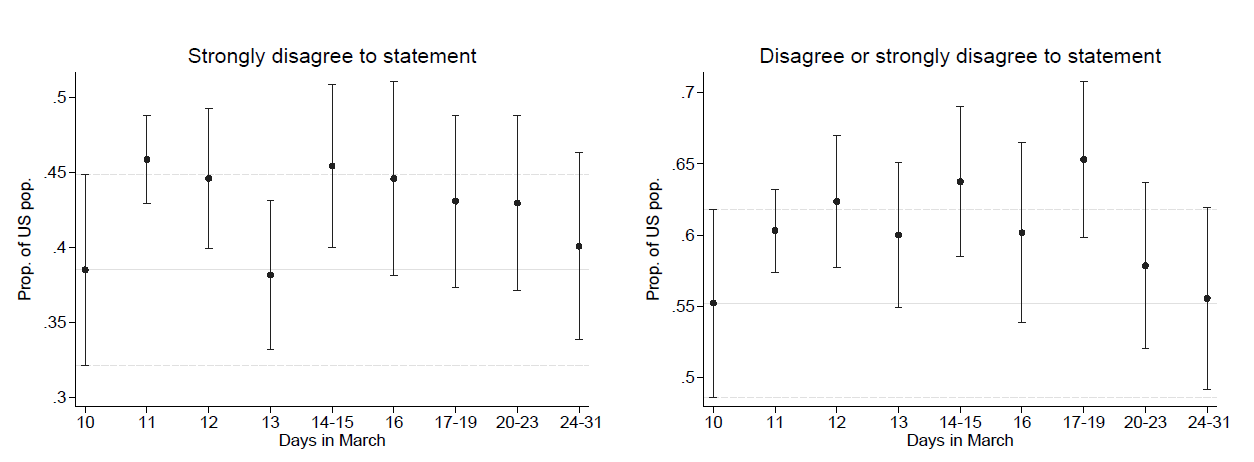

Supplement: S1 Fig — Notes: Source of Data: “Understand America Study” (UAS), surveys 121 and 230. UAS survey 121 was fielded between January 2018 and March 2020 and survey 230 was collected between March 10 and March 31, 2020. Plot on the left shows weighted proportions of the US population, along with 95% confidence intervals, that strongly disagree to the statement: “I see myself as someone who is depressed, blue”. Plot on the right shows weighted proportions of the US population that strongly disagree or disagree to the same statement. We use post-stratification weights so that the weighted means are representative of the US population for each particular time period on the x–axis. (TIF) [file pone.0241895.s001.tif]
