# Supplementary material for: Predictors of mental health during the Covid-19 pandemic in the US: Role of economic concerns, health worries and social distancing
Source: PLoS One. 2020 Nov 11;15(11):e0241895. doi: 10.1371/journal.pone.0241895 (PMC7657497; doi:10.1371/journal.pone.0241895)
Supplement: S1 Appendix — (PDF) [file pone.0241895.s002.pdf]

S1 Appendix. Understanding America Study (UAS) UAS is a study that is supported by the Social Security Administration (SSA) and the National Institute on Aging (NIA). It has since 2014 collected more than 230 online surveys on various topics, ranging from cognitive abilities, environment, consumer behavior and politics to name but a few, for which the data is publicly available [17]. The UAS uses address-based sampling with sequential sample batching, where addresses were drawn from the Computerized Delivery Sequence (CDS) file created by the U.S. Postal Service. Annual attrition rates are modest (on the order of 8-9% per year).

The provision of tablets and free Internet to households without prior Internet access solves a coverage problem faced by convenience Internet panels. Respondents without prior Internet access have a very different demographic and socio-economic profile than respondents with Internet access such as they are more likely to have low incomes and education, to be non-white, less health and older (70+ years). However, among the non-Internet households, the probability of signing up for UAS is not related to these background characteristics, expect for age. One may interpret these findings as an indication that with respect to the demographics studied, the recruited non-Internet households are representative of the part of the population without Internet (except possibly with respect to age) [17].
