# Supplementary material for: Predictors of mental health during the Covid-19 pandemic in the US: Role of economic concerns, health worries and social distancing
Source: PLoS One. 2020 Nov 11;15(11):e0241895. doi: 10.1371/journal.pone.0241895 (PMC7657497; doi:10.1371/journal.pone.0241895)
Supplement: S2 Appendix — (PDF) [file pone.0241895.s003.pdf]

S2 Appendix. The PhQ-4 and Covid-19-related questions The PhQ-4 is composed of the following four questions: *Over the last two weeks, how often have you been bothered by any of the following problems?*

- Feeling nervous, anxious, or on edge
- Not being able to stop or control worrying
- Feeling down, depressed, or hopeless
- Little interest or pleasure in doing things

Possible answers to these questions were: *0 Not at all, 1 Several days, 2 More than half the days, 3 Nearly every day.* We added up the answers to these four questions and

computed a PhQ-4 score, ranging from 0 to 12. We followed [26] and categorized respondents as having *no depression/anxiety symptoms* if their score equaled to 0, 1 or 2, *mild depression/anxiety symptoms* if their score ranged from 3 to 5, *moderate depression/anxiety symptoms* if their score ranged from 6 to 8 and *severe depression/anxiety symptoms* if their score ranged from 9 or above.

The question on the probability of being infected was phrased as: *"On a scale of 0 to 100 percent, what is the chance that you will get the coronavirus in the next three months? If you're not sure, please give your best guess."* The one on the probability of dying if infected was phrased as: *"If you do get the coronavirus, what is the percent chance you will die from it? If you're not sure, please give your best guess."* Finally, the question about the probability of running out of money because of the Covid-19 pandemic was: *"The coronavirus may cause economic challenges for some people regardless of whether they are actually infected. What is the percent chance you will run out of money because of the coronavirus in the next three months?"*
