# Supplementary material for: Predictors of mental health during the Covid-19 pandemic in the US: Role of economic concerns, health worries and social distancing
Source: PLoS One. 2020 Nov 11;15(11):e0241895. doi: 10.1371/journal.pone.0241895 (PMC7657497; doi:10.1371/journal.pone.0241895)
Supplement: S3 Appendix — (PDF) [file pone.0241895.s004.pdf]

S3 Appendix. **γ-standardization of the continuous variables** The coefficient of standardized continuous variable can be interpreted as the effect of a one standard deviation increase in that variable on the outcome of interest. On the other hand, the coefficient associated to a dichotomous variable corresponds to a marginal increase in that variable from 0 to 1, which is usually larger in magnitude than a standard deviation increase in the continuous variable. [48] therefore suggests to rescale the continuous variable by more than just their standard deviation so that the marginal effect of the continuous variables can be more meaningfully compared to the marginal increase in the dichotomous variable.

In essence, following the notation in the main text, his idea is to choose γ in such a way that the $\gamma\times$ standard deviation increase in the continuous variables correspond to the marginal increase in the dichotomous variable from 0 to 1. One can therefore use the dichotomous variable characteristic as a benchmark for rescaling the continuous variables. [48] suggests to use γ =2, which works well when the mean of the dichotomous variable is close or equal to 0.5. Indeed, if the mean of a dichotomous variable is equal to 0.5, then its standard deviation would be equal to 0.5 $(= \sqrt{0.5 \times (1 - 0.5)})$. A two standard deviation increase in the continuous variable

would therefore correspond to a marginal increase in the dichotomous variable from 0 to 1, as 1 is equal to two standard deviations of the dichotomous variables as well.

In our study, because the mean of social distancing is equal to 0.655, we choose $\gamma = 2.103$. Indeed, $2.103\times$ the standard deviation of social distancing is equal to 1 ($\cong 2.103 \times \sqrt{0.655 \times (1 - 0.655)}$) so that a $\gamma\times$ standard deviation increase in the continuous variables corresponds to an increase in the dichotomous variable from 0 to 1. The coefficients associated with the transformed continuous variables can then be meaningfully compared to the coefficient associated with the dichotomous variable.
