# Supplementary material for: Predictors of mental health during the Covid-19 pandemic in the US: Role of economic concerns, health worries and social distancing
Source: PLoS One. 2020 Nov 11;15(11):e0241895. doi: 10.1371/journal.pone.0241895 (PMC7657497; doi:10.1371/journal.pone.0241895)
Supplement: S1 Table — (PDF) [file pone.0241895.s005.pdf]

S1 Table. Descriptive statistics - additional variables

	Pop. mean	Pop. std. deviation
Male	0.489	0.500
Age	48.429	16.583
Less than high school	0.086	0.280
High school graduate or GED	0.297	0.457
Some college (no degree)	0.277	0.447
College graduate and above	0.341	0.474
White	0.772	0.420
Black	0.126	0.332
Other races	0.102	0.302
Currently married	0.557	0.497
Hardware (internet-connected tablet) provided	0.044	0.205

Notes: Data source: “Understand America Study” (UAS), survey 230, collected between March 10 and March 31, 2020. We use sample weights to make the survey representative of the U.S. population aged 18 and older. “Pop.” is short for population and “std.” is short for standard.
