# Supplementary material for: Predictors of mental health during the Covid-19 pandemic in the US: Role of economic concerns, health worries and social distancing
Source: PLoS One. 2020 Nov 11;15(11):e0241895. doi: 10.1371/journal.pone.0241895 (PMC7657497; doi:10.1371/journal.pone.0241895)
Supplement: S2 Table — (PDF) [file pone.0241895.s006.pdf]

S2 Table. Differences between UAS responders and non-responders

	Population means			
	Responders	Non-responders	χ^2	p-value
Male	0.489	0.463	1.926	0.165
Age	48.448	47.797	0.965	0.326
Less than high school	0.084	0.096	1.107	0.293
High school graduate or GED	0.296	0.299	0.015	0.901
Some college (no degree)	0.277	0.279	0.018	0.894
College graduate and above	0.342	0.326	0.871	0.351
White	0.773	0.759	0.703	0.402
Black	0.125	0.138	0.938	0.333
Other	0.102	0.103	0.004	0.947
Currently married	0.557	0.512	5.659	0.017

Notes: Source of Data: "Understand America Study" (UAS), survey 230, collected between March 10 and March 31, 2020. We use sample weights to make the survey representative of the U.S. population aged 18 and older. Difference in age was assessed using a t-test and the number in column 4 corresponds to the F-value.
