# Supplementary material for: Predictors of mental health during the Covid-19 pandemic in the US: Role of economic concerns, health worries and social distancing
Source: PLoS One. 2020 Nov 11;15(11):e0241895. doi: 10.1371/journal.pone.0241895 (PMC7657497; doi:10.1371/journal.pone.0241895)
Supplement: S3 Table — (PDF) [file pone.0241895.s007.pdf]

S3 Table. Associations with Covid-19 cases and related number of deaths and mental health score (PhQ-4) - Average marginal effects

	PhQ-4 categories			
	None	Mild	Moderate	Severe
Number of cases in US (log)	-0.026***	0.012***	0.007***	0.007***
	[-0.040,-0.013]	[0.006,0.018]	[0.004,0.010]	[0.003,0.012]
Number of deaths in US (log)	-0.031***	0.014***	0.008***	0.009***
	[-0.046,-0.015]	[0.007,0.021]	[0.004,0.012]	[0.004,0.013]

Notes: Average marginal effects resulting from weighted ordered probit regressions with robust confidence intervals clustered at the state level in brackets, * $p < 0.1$, ** $p < 0.05$, *** $p < 0.01$. The number of cases and deaths in the US is transformed using log. Specifications include state fixed effects. The list of control variables includes: sex, age and age^2, educational level (binary variable for each category), race and whether the respondent was married at the time of the interview. We control for depression level characteristics prior to the Covid-19 pandemic, along with the year and month when this measure was collected. We use sample weights to make the survey representative of the U.S. population aged 18 and older. Data source: "Understanding America Study" (UAS) collected between March 10 and March 31, 2020.
