# Supplementary material for: Predictors of mental health during the Covid-19 pandemic in the US: Role of economic concerns, health worries and social distancing
Source: PLoS One. 2020 Nov 11;15(11):e0241895. doi: 10.1371/journal.pone.0241895 (PMC7657497; doi:10.1371/journal.pone.0241895)
Supplement: S4 Table — (PDF) [file pone.0241895.s008.pdf]

S4 Table. Associations with mental health score (PhQ-4) - Average marginal effects

	PhQ-4 categories			
	None	Mild	Moderate	Severe
Perceived risk of running out of money (std)	-0.111***	0.051***	0.030***	0.031***
	[-0.140,-0.083]	[0.037,0.064]	[0.022,0.038]	[0.022,0.039]
Perceived risk of dying from Covid-19 (std)	-0.046***	0.021***	0.012***	0.013***
	[-0.070,-0.022]	[0.010,0.032]	[0.006,0.019]	[0.006,0.019]
Self-reported social distancing	-0.068***	0.031***	0.018***	0.019***
	[-0.097,-0.038]	[0.017,0.044]	[0.010,0.027]	[0.010,0.027]

Notes: Average marginal effects resulting from weighted ordered probit regressions with robust confidence intervals in brackets, * $p < 0.1$, ** $p < 0.05$, *** $p < 0.01$. Specifications include state and time fixed effects. The list of control variables includes: sex, age and age^2, educational level (binary variable for each category), race and whether the respondent was married at the time of the interview. We control for depression level characteristics prior to the Covid-19 pandemic, along with the year and month when this measure was collected. We use sample weights to make the survey representative of the U.S. population aged 18 and older. "Perceived risk of running out money" and "Perceived risk of dying from Covid-19" have been standardized by subtracting their respective mean and dividing by $\gamma\times$ their standard deviations. Data source: "Understanding America Study" (UAS) collected between March 10 and March 31, 2020.
