# Supplementary material for: Predictors of mental health during the Covid-19 pandemic in the US: Role of economic concerns, health worries and social distancing
Source: PLoS One. 2020 Nov 11;15(11):e0241895. doi: 10.1371/journal.pone.0241895 (PMC7657497; doi:10.1371/journal.pone.0241895)
Supplement: Attachment — Submitted filename: Review_Covid 19.docx [file pone.0241895.s010.docx]

Title: Predictors of mental health during the Covid-19 pandemic in the US: role of economic concerns, health worries and social distancing

Review

- I think this article timely in the context of Covid-19 pandemic.
- The article demonstrated strongly association between concerns about the economic consequences of the pandemic and worsening mental health. If we assumed a reverse causality which means that the person who have worsening mental health might be likely to be a lower income person, the association among them might be overestimated.
- The Authors showed association between social distance and worsening mental health. If we assumed a reverse causality which means that worsening mental health might increase social distance, the association among them might be overestimated.
- I suggest the authors to describe the reverse causality from outcome variable to the control variables in detail.
